# Nucleophosmin 1 associating with engulfment and cell motility protein 1 regulates hepatocellular carcinoma cell chemotaxis and metastasis

**DOI:** 10.1515/med-2023-0708

**Published:** 2023-05-25

**Authors:** Gangqi Yang, Hongyan Li, Zheng Dong, Kai Deng, Yinying Lu

**Affiliations:** Department of Infectious Diseases, The Affiliated Hospital of Guizhou Medical University, Guiyang, Guizhou 550001, China; General Surgery Department and Neurology Department, Xuanwu Hospital, National Clinical Research Center for Geriatric Diseases, Beijing 100053, China; Comprehensive Liver Cancer Center, 5th Medical Center of the PLA General Hospital, Beijing 100039, China; Guangdong Key Laboratory of Epigenetics, College of Life Sciences and Oceanography, Shenzhen University, Shenzhen, Guangdong 518055, China; The First Affiliated Hospital of Chongqing Medical College, Chongqing 400016, China

**Keywords:** NPM1, ELMO1, chemotaxis, metastasis, hepatocellular carcinoma, DMF

## Abstract

The chemokine, C-X-C motif chemokine ligand 12 (CXCL12) and its G-protein-coupled receptor (GPCR) and C-X-C chemokine receptor type 4 (CXCR4), are closely associated with promoting hepatocellular carcinoma (HCC) chemotaxis and metastasis. The binding of CXCL12 and CXCR4 depends on the heterotrimeric Gi proteins to regulate actin polymerisation and mobility in HCC. Although the role of GPCR/Gi signalling in carcinogenesis migration has been intensively studied, the detailed mechanism remains largely unknown. In this study, a small interfering RNA technique was used to knock down the Nucleophosmin 1 (NPM1) gene expression. Through the chemotaxis and invasion assays, wound healing, proliferation, filamentous-actin, immunofluorescence, immunohistochemical assays, and co-immunoprecipitation assays, we investigated the specific biological role and underlying mechanisms of the NPM1 in HCC. Additionally, dimethyl fumarate (DMF), a fumaric acid ester, was used to inhibit the HCC cell chemokines and metastasis by regulating ELMO1 and NPM1. Therefore, this study reported that NPM1 gene expression was upregulated in the HCC tissues and cell lines. The NPM1 knockdown significantly inhibited the proliferation, migration, and chemotaxis of the HepG2 cells *in vitro.* Further mechanistic studies suggested that the NPM1 interacts with ELMO1 and the CXCL12/CXCR4 pathway activates NPM1-dependent regulation of the ELMO1 localisation. Furthermore, the DMF significantly inhibited tumour metastasis induced by the NPM1/ELMO1 signalling pathway, as observed in *in vitro* cell functional experiments. These data suggested that as a potentially novel therapeutic approach, the simultaneous targeting of NPM1 and ELMO1 could effectively be used to treat HCC.

## 1 Introduction

The incidence of hepatocellular carcinoma (HCC) in China has steadily increased recently; it is one of the most prevalent digestive system malignancies, with morbidity and mortality ranking sixth and fourth among all malignant tumours, respectively, according to the global cancer statistics in 2018 [1]. Early-stage patients with HCC have relatively favourable treatment, including liver transplantation and surgical resection, with a 5 year survival rate of 75% [2]. Unfortunately, most patients are diagnosed with HCC at an advanced stage, which leads to high mortality [3]. Therefore, there is an urgent need to investigate the underlying molecular mechanisms of HCC metastasis and identify more sensitive diagnostic biomarkers for HCC.

Nucleophosmin 1 (NPM1), also known as B23, numatrin, or NO38, is a highly abundant multifunctional nucleolar phosphoprotein comprising 294 amino acids [4]. It shuttles between the nucleus and the cytoplasm, where it is involved in several important biological functions, such as ribosome biogenesis, chromatin remodelling, centrosome duplication, embryogenesis, apoptosis, and DNA repair [5]. Most of these functions are mediated through the interactions with various protein partners. Previous studies have primarily focused on the oncogenic role of NPM1 in acute myeloblastic leukaemia (AML). Additionally, NPM1 is one of the most commonly mutated genes in AML [6]. Many studies have reported that mutations and rearrangements in NPM1 are found in 35% of patients with AML, and NPM1 mutational persistence significantly affects the relapse rate and might be more resistant to chemotherapy in leukaemia [7]. Therefore, NPM1 could be used as a biomarker and therapeutic target for haematological malignancies. In contrast to AML, studies on the role and function of NPM1 in solid tumours are rare; however, NPM1 is frequently overexpressed in various cancer cells [8]. In the present reports of bladder cancer, decreased protein-coding NPM1 transcript levels supported the association of Myc gene expression, aberrant activation of the β-catenin/c-Myc, and the AFF4/NF-κB/Myc signalling pathways to regulate cell activities and promote cancer migration in bladder cancer [9]. In addition, high NPM1 expression is linked with tumourigenesis in lung adenocarcinoma and colon cancer [10,11]. Therefore, these studies show that the value of NPM1 requires investigation in further studies on different tumours.

Additionally, heterotrimeric G proteins are guanine nucleotide binding proteins, which represent a family of intercellular molecular transducers stimulated by several extracellular agents such as chemokines, neurotransmitters, and hormones. The G proteins are typically represented by the evolutionarily conserved plasma membrane-bound proteins, which act as intracellular signalling molecules through activating the associated G-protein coupled receptors (GPCRs). Heterotrimeric G proteins are recognised to consist of three major subunits as follows: alpha (α), beta (β), and gamma (γ) [11,12]. Interestingly, the cellular function and dynamic regulation of G proteins have been extensively investigated over the past two decades. The dissociation of heterotrimeric G-proteins is induced by activating a chemokine receptor, which successively activates the intracellular signal transduction pathways to regulate the filamentous-actin (F-actin) cytoskeleton and mediate cell migration and metastasis [13–17].

Furthermore, metastasis is a consecutively linked process that describes the spread of cancerous cells from the original tumour site, migrating through the bloodstream or the lymph system and other pathways to other major organs or tissues [18]. Chemokines are a superfamily of chemotactic and pro-inflammatory proteins which can regulate multiple cellular processes, such as adhesion, migration, and metastasis. Moreover, cancer cell metastasis is enhanced by chemotaxis, which is a movement of an organism or cell in response to chemical signals in the environment. Notably, in tumour cells, the binding of the chemokine to its ligand, a subfamily of the GPCR and the guanine exchange factor (GEF) activity, promotes the release of guanosine diphosphate (GDP) from the Gαi subunit and subsequent binding of guanosine triphosphate (GTP), which results in cancer cell migration from the liver to the metastatic cancer sites [19]. Signalling by the C-X-C chemokine receptor type 4 (CXCR4) promoted by its cognate ligand, C-X-C motif chemokine ligand 12 (CXCL12), also known as stromal-cell derived factor-1alpha, or SDF-1 alpha, plays a major critical role in cell chemotaxis and F-actin polymerisation, which initially control cell migration in various cancer types. For example, in a mouse model of breast cancer with lung metastasis, the chemokine receptor, CXCR4, and its ligand, CXCL12, control the metastasis of the breast cancer cells, where the expression of CXCL12 is high in breast cancer cells [20–22]. Although they are linked with cancer migration and metastasis, the detailed mechanisms by which chemokines and their receptors exert their effects remain largely unknown.

In many CED-12 homologs, such as mammals, Dictyostelium, and Caenorhabditis, the ELMO protein family acts as GEFs for activating the Rac protein, which is necessary for controlling actin cytoskeleton and cell migration and engulfment [23]. At present, many research works have reported that the family members of the ELMO protein play a prominent role in the progression of different kinds of cancer. The report demonstrated GPCR binding to the chemokine triggers a direct association between Gβγ subunits and the ELMO/Dock180 complex to regulate cancer cells motility [24,25]. In human breast cancer, it is the first time that ELMO1/DOCK180 and Rac molecular synergistically activate the processes of breast cancer cells chemotaxis and metastasis [26]. In glioma carcinoma cells, IL-8 stimulates ELMO1-NF-κB-Snail signalling to drive the epithelial-mesenchymal transition [27]. Overexpression of ELMO1 has a significant influence on cancer cell migration and invasion in the current study of hepatocellular carcinoma [28]. Therefore, we can fully understand the value of ELMO1, which regulates cancer metastasis and requires investigation in further studies on solid tumours. Our previous study reported that ELMO1 and its interacting protein Annexin A2 are involved in the CXCL12/CXCR4 signalling pathway to regulate the chemotaxis and metastasis of HCC cells [29]. Thus, it is reasonable to speculate that NPM1, functioning as a new ELMO1-interacting protein, also could promote HCC chemotaxis and migration through the CXCL12/CXCR4 signalling pathway. Moreover, DMF is a prescribed oral therapy for multiple sclerosis, which exhibits antitumour activities. Previous reports reported that DMF is critical for inhibiting melanoma and colon cancer cell growth and metastasis [30,31]. Our study also explores whether dimethyl fumarate (DMF) might be a potential anticancer drug for HCC metastasis by targeting the NPM1/ELMO1. To validate the hypothesis mentioned above, we characterised that NPM1 interacts with ELMO1 and further examined its function in HCC. Our data indicate that the altered localization of the NPM1 has a significant effect on CXCL12-mediated HCC cell chemotaxis, migration, and invasion by binding to different protein partners. Additionally, DMF synergistically suppressed HCC progression through the NPM1/ELMO1 signalling pathway, which suggests that simultaneously targeting NPM1 and ELMO1 for HCC metastasis treatment is a promising approach to increase its therapeutic effects.

## 2 Methods

### 2.1 Cell culture and transient transfection

The human liver cancer cell line, HepG2, was obtained from the American Type Culture Collection (ATCC) and cultured in Gibco Dulbecco’s Modified Eagle Medium (DMEM) (Gibco Invitrogen Corporation, Australia) supplemented with 10% foetal bovine serum (FBS, Gibco Invitrogen Corporation, Australia). All the cells were maintained in a controlled humidified atmosphere containing 5% of carbon dioxide and 95% of air. Therefore, to determine whether silencing NPM1 affects the HepG2 cell chemotaxis and migration, three different specific small interfering RNAs (siRNAs) targeting NPM1 RNA and a negative control (NC) siRNA were synthesised by GenePharma (Shanghai, China) and used for *in vitro* transfection. Finally, the HepG2 cells were incubated for 48 h, and the protein expression was subsequently confirmed by Western blotting.

### 2.2 Exogenous co-immunoprecipitation (Co-IP)

Briefly, HepG2 cells seeded in 6-well plates were plated to 60–70% confluency before infection. The goal was to obtain a high expression level of NPM1 and ELMO1 proteins by transfecting GV141 Flag-ELMO1. Total protein was extracted from the transfected cells using ice-cold RIPA buffer containing phenylmethylsulfonyl fluoride (PMSF). First, whole-cell lysates were immunoprecipitated overnight with anti-flag monoclonal and rabbit IgG antibodies as controls. Then, 30 µL of Dynabeads Protein A/G magnetic beads were added to the immune complex and continuously mixed. Next the precipitates were washed four times with lysis buffer and eluted from the magnetic beads by boiling with SDS sample buffer.

### 2.3 Immunofluorescence assay

Briefly, the cells were seeded on round glass coverslips in 24-well plates at a density of 1 × 10^5^ cells/well. First, the cells were starved in a serum-free medium for 3 h and stimulated with CXCL12 (100 ng/mL). Next cells firmly attached to the coverslips were washed three times in a phosphate-buffered saline (PBS), fixed in 4% paraformaldehyde for 15 min, and permeabilised with PBS containing 0.2% Triton X-100 for 20 min. Next, the cells were blocked with 10% donkey serum at room temperature for 30 min and then incubated overnight with the primary antibody at 4°C. Subsequently, the cells were stained with anti-rabbit Alexa Fluor 488-conjugated or anti-mouse 546-conjugated secondary antibody in the dark and imaged using a conventional wide-field fluorescence microscope (×100).

### 2.4 Chemotaxis and invasion assay

Firstly, transwell chambers with 8 μm pore size were coated with 60 μL of Matrigel in invasion assay, but not in chemotaxis assay. Next cells were suspended and seeded into the upper chambers with an 8 µm microporous filter. In contrast, serum-free DMEM solution containing the chemokine (0, 10, 100, and 1,000 ng/ml CXCL12) was placed in the bottom chambers. After incubation for 24 h, the non-migrated cells were washed off, and the membrane was stained with crystal violet and counted under a light microscope.

### 2.5 Wound healing assay

A wound-healing assay was performed to evaluate the tumour cell mobility *in vitro*, and cells were plated in 6-well plates at a density of 5 × 10^5^ cells/well and grown until they reached 80–90% confluency. After transfection with the specific siRNA for 24 h, the confluent cell monolayer was scratched using a sterile plastic 10 μL pipette tip and washed three times with PBS. The distance between the wounds at 0, 4, 8, 12, and 24 h was observed and measured under a light microscope (×400).

### 2.6 Cell proliferation assay

After 24 h of transfection, the liver cancer cells and HepG2 cells (4 × 10^3^ cells/well) were seeded in 96-well culture plates. Next cell proliferation was measured using the Cell Counting Kit-8 (CCK8) method. After 24, 48, and 72 h of incubation, cell proliferation was evaluated by adding 10 µL CCK8 solution to each well and incubated for 4 h at 37°C.

### 2.7 Immunohistochemical assay

We performed an immunohistochemical assay for the entire sample (70 HCC tissues and 39 paracancerous tissues) from the Department of Liver Cancer, Beijing 302 Hospital. Cells were measured semi-quantitatively. The primary antibody NPM1 (1:1,000) was used for incubation, and the detection was conducted using a horseradish peroxidase (HRP)-conjugated secondary antibody and DAB chemistry kit. The statistical method used was the chi-square (*χ* ^2^) statistics.

### 2.8 Statistical analysis

Statistical analysis was performed using GraphPad Prism 8.0 (GraphPad Software, San Diego, CA, United States) and SPSS 22.0 software and chi-square (*χ* ^2^) statistics. In all statistical analyses, *p* values <0.05 were considered significant.


**Ethical statement:** The studies involving human participants were reviewed and approved by the Ethics Committee of the Fifth Medical Center of Chinese PLA General Hospital. The patients/participants provided their written informed consent to participate in this study.

## 3 Result

### 3.1 High expression of NPM1 in the metastatic HCC cells

**Figure 1:**
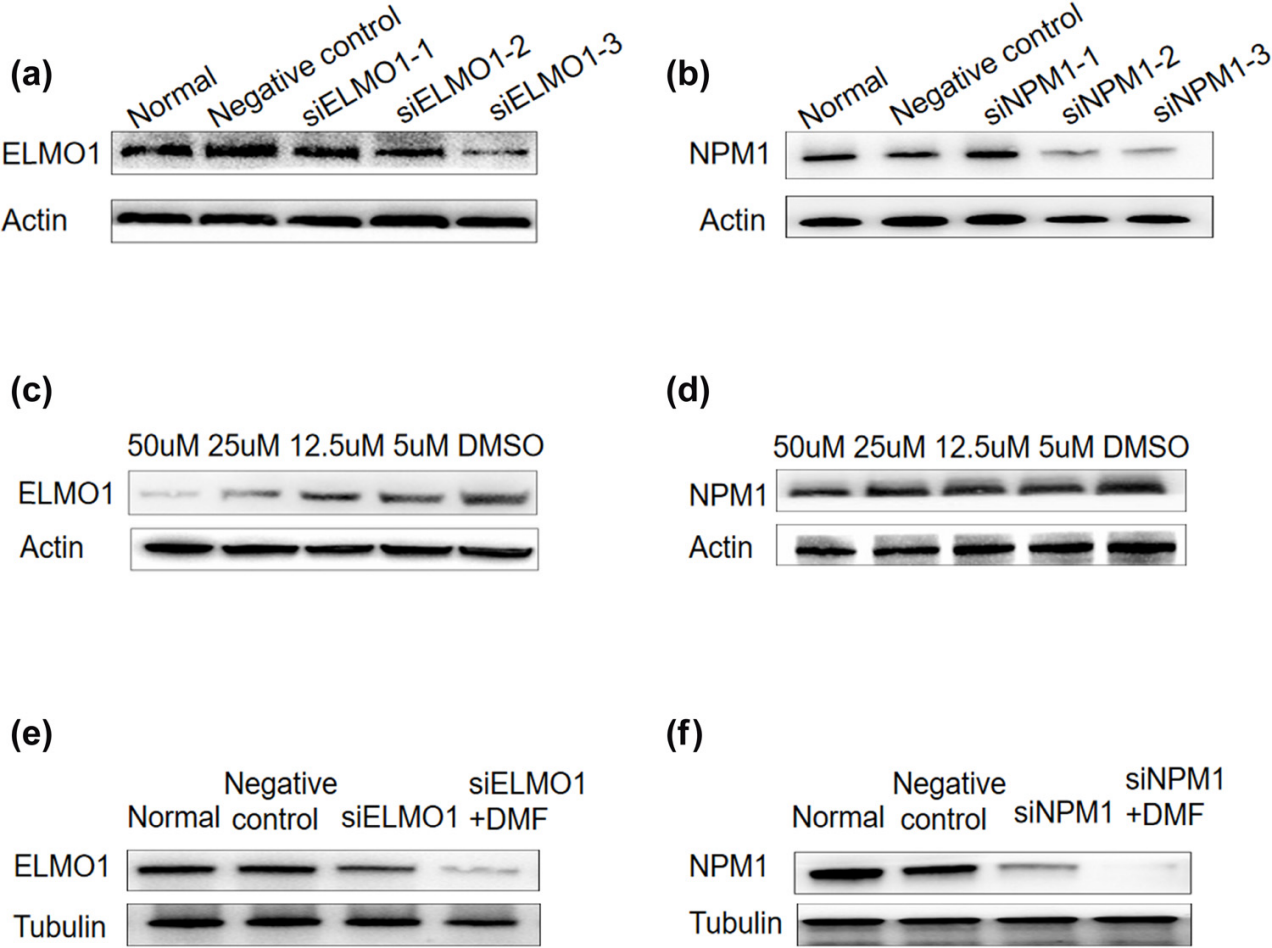
The expression of NPM1 and ELMO1 in HCC. (a and b) Western blot analysis of siRNA-mediated knockdown of NPM1 and ELMO1 protein expression in HCC cell lines. Actin was used as a loading control. (c and d) The expression of NPM1 and ELMO1 were detected by western blotting in hepatocellular carcinoma cells with different concentrations of DMF treatment. (e and f) HepG2 cells were treated with siNPM1 and siELMO1 with or without DMF for 24 h. NPM1 and ELMO1 were examined by western blot assay (Data are the mean value of three independent experiments; two-way ANOVA, ***p < 0.001.).

To elucidate the effect of NPM1 protein on the developing migration and aggression of the HCC cell line HepG2, we used the siRNA method to knock down NPM1 or ELMO1 expression in HCC cells. Our results initially indicated that protein levels of NPM1 and ELMO1 were relatively high in the metastatic HCC cell (Figure 1a and b). Then, to validate the effect of DMF treatment on HCC cell migration via NPM1 and ELMO1 proteins, we first detected the expression of NPM1 and ELMO1 using Western blotting after HepG2 cells were treated with varying concentrations of DMF. Western blotting results demonstrated that NPM1 and ELMO1 expression levels decreased dose-dependently, and 50 µM DMF had the strongest inhibitory effect (Figure 1c and d). Additionally, HepG2 cells were treated with siNPM1 or siELMO1 combined with 50 µM DMF and the same trend was observed (Figure 1e and f). Therefore, 50 µM DMF was determined to be the suitable concentration for subsequent cell functional experiments.

### 3.2 Role of NPM1 in the migration and chemotaxis of HCC cells

**Figure 2:**
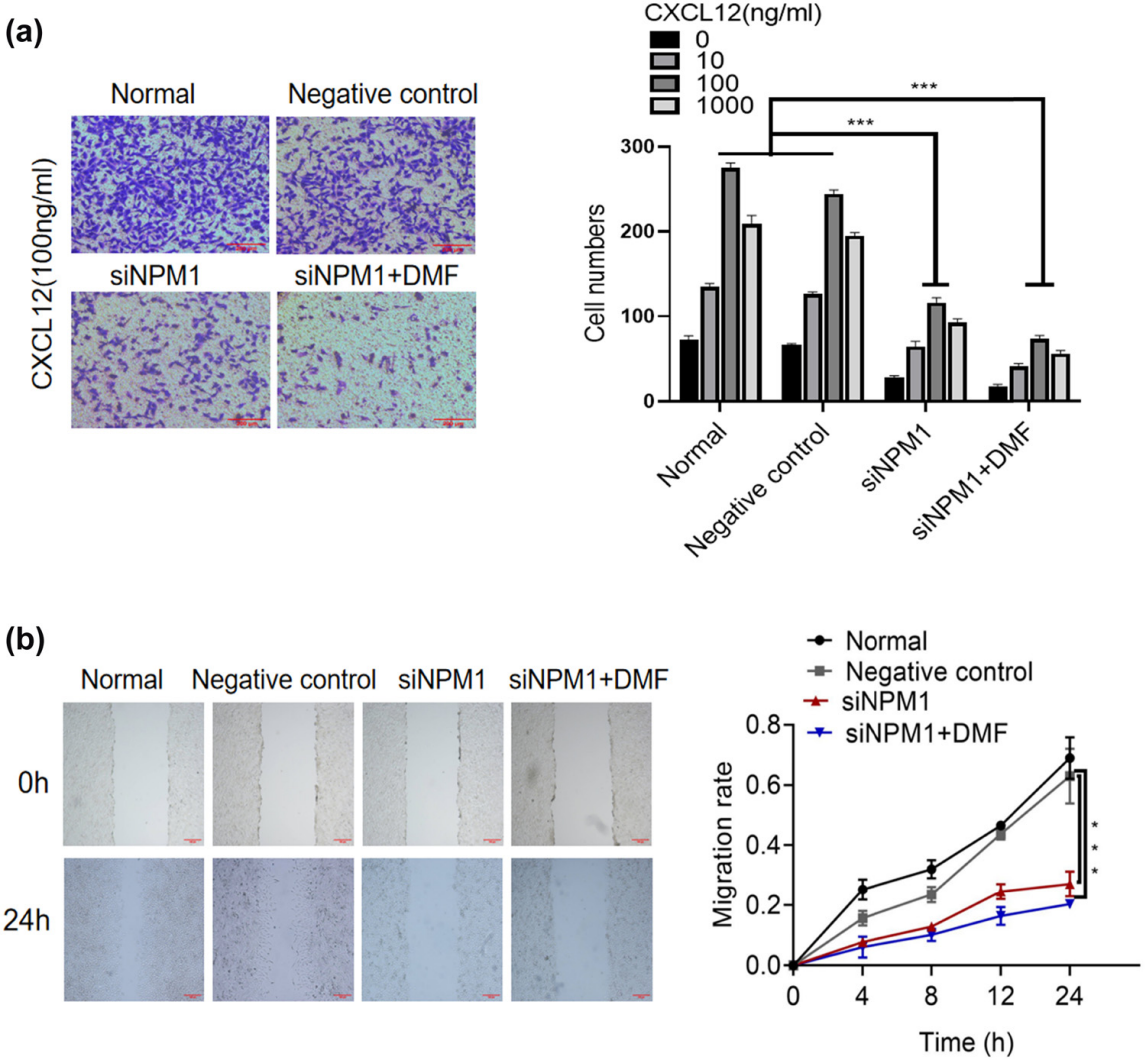
The function of NPM1 in hepatocellular carcinoma chemotaxis and migration. (a) Chemotaxis analysis in siRNA knockdown cells (×200). (b) Wound healing scratch assay in siNPM1 cells: 2 × 10^5^ cells were plated in 6-well plates and formed the cell monolayer using a pipette tip. The wound gap distance was measured at 0, 6, 12, 18, and 24 h (×100) (Data are the mean value of three independent experiments; two-way ANOVA, *** *p* < 0.001.).

Then, a cell chemotaxis assay was performed, and the results showed that siRNA-mediated reduction in NPM1 significantly decreased CXCL12-induced number of chemotactic HCC cells (Figure 2a). In addition, the wound healing assay showed that the migration capacity of siNPM1 cells was lower than that of the blank and NC groups (Figure 2b). These results also validated that compared with siNPM1 group, siNPM1 + DMF treatment group could further inhibit cell chemotaxis (Figure 1c) and migration (Figure 1d) in HCC.

### 3.3 NPM1 silencing inhibited invasion, proliferation, and F-actin polymerisation assays of the HCC cells

**Figure 3:**
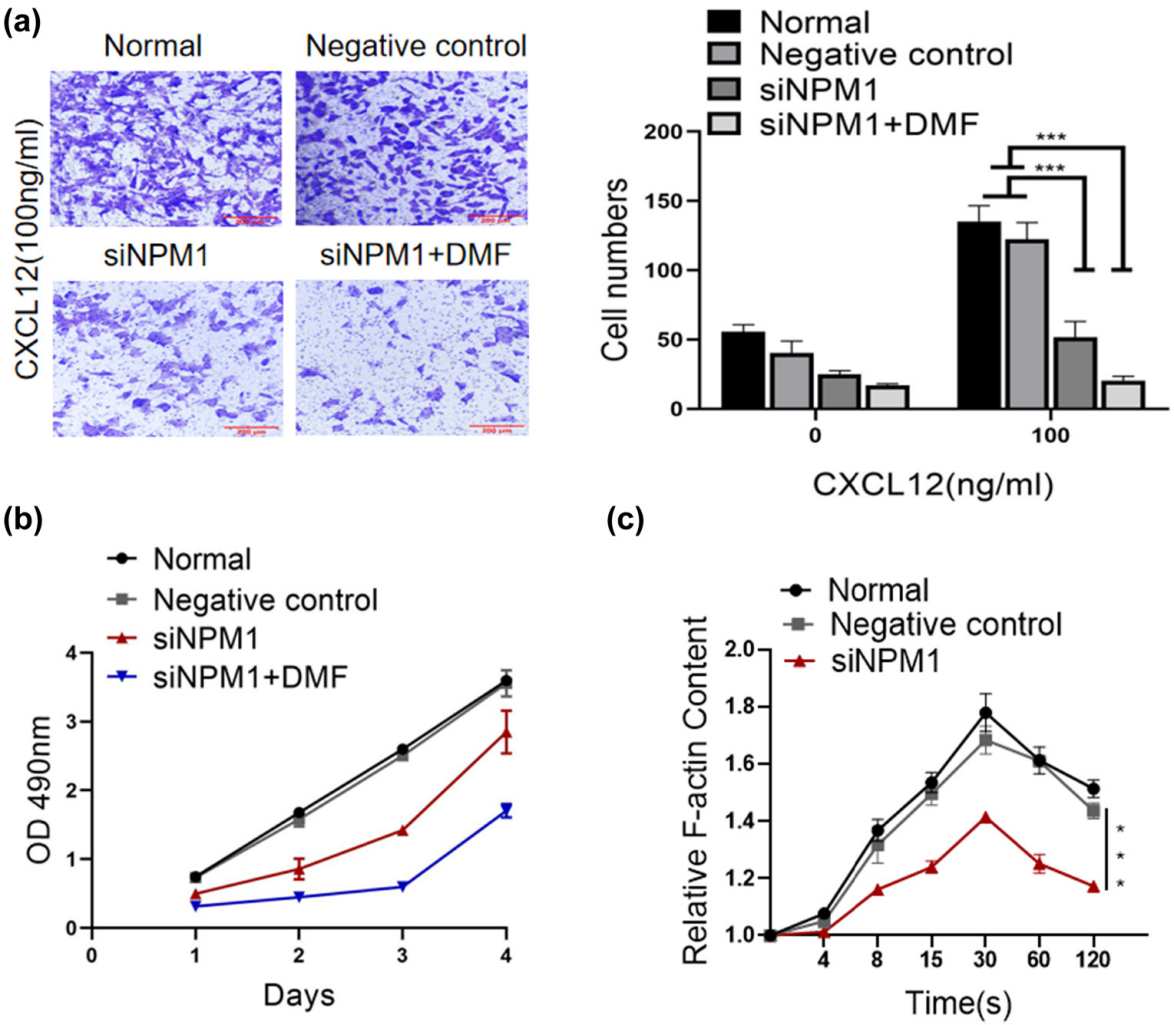
NPM1 knockdown inhibited the proliferation and invasion of HepG2 cells. (a) Matrigel invasion assay and quantification analysis showed that the knockdown of NPM1 significantly inhibited the CXCL12-mediated invasive abilities of HepG2 cells (×200). (b) Proliferation assay for HepG2 cells with or without NPM1 downregulated expression. The cells were plated at low densities (1%, 5 × 10^3^ cells per 35 mm dish). The proliferation rate of siNPM1 cells was much higher than that of normal cells. (c) NPM1 knockdown could inhibit F-actin polymerisation of HepG2 cells. Statistical analysis was conducted for the F-actin value at different time points (0, 4, 8, 15, 30, 60, and 120 s). Time course of fold increased relative F-actin content in normal, control, siNPM1 cells, and siNPM1 combined with DMF in response to CXCL12 stimulation (Data are the mean value of three independent experiments; two-way ANOVA, ***p < 0.001.).

Furthermore, we detected the potential role of NPM1 in the invasive ability in HCC cells. The results showed that NPM1 knockdown by siRNA repressed CXCL12-induced invasion of the HepG2 cells, while CXCL12-induced invasion was almost completely blocked by siNPM1 combined with DMF treatment of HepG2 cells for 24 h (Figure 3a). We also evaluated whether NPM1 affected cell proliferation. The cell adhesion assay data were similar to the transwell assay which exhibited that the downregulated NPM1 group weakened HepG2 cells proliferation, and the siNPM1 combined with DMF group showed the weakest proliferation ability (Figure 3b). In cancer cells, chemokine binding to receptors is a key regulator of actin polymerisation and transient F-actin assembly, ultimately enhancing cell migration and invasion. Consistent with a previous report, we found that CXCL12 caused a significant transient accumulation of intracellular F-actin levels in HepG2 cells, which peaked within 30 s (Figure 3c). In summary, our findings demonstrate that NPM1 can promote the proliferation, chemotaxis, migration, and invasive capacity of the HCC cells, and that DMF inhibits the HCC cell mobility by downregulating NPM1/ELMO1 expression.

### 3.4 NPM1 interacts with ELMO1

**Figure 4:**
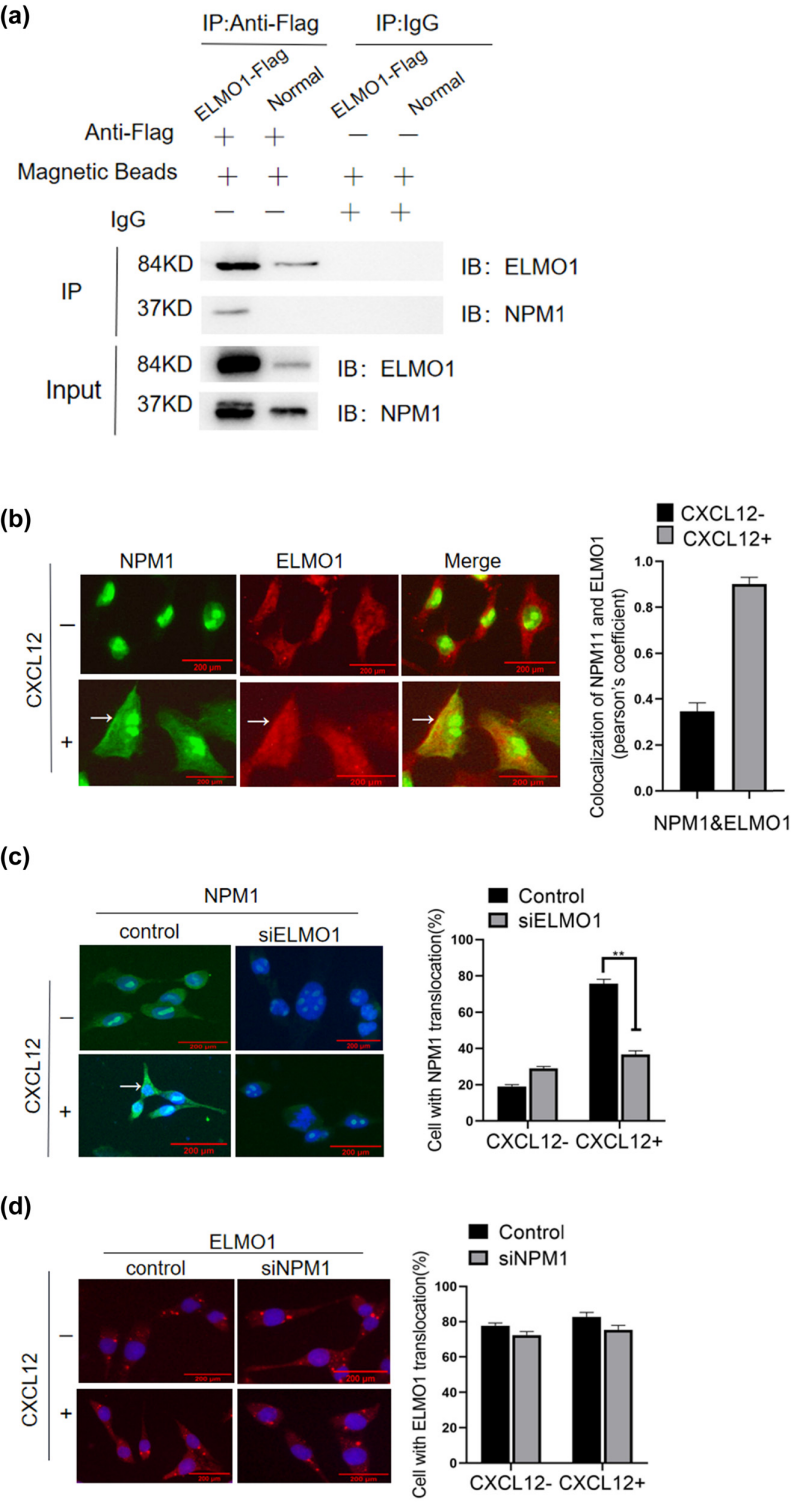
NPM1 interacts with ELMO1. (a) Co-IP assay of NPM1-ELMO1interaction. The precipitated proteins were separated on SDS-PAGE and were probed with NPM1 and ELMO1 antibodies. (b) Cells were observed under a fluorescence microscope (×200). Upon stimulation with CXCL12, both the plasma membrane and the cytoplasm localization of NPM1 and ELMO1 were evident. Qualification of the colocalization extent was calculated with Image J software. (c) The translocation of NPM1 to the cell plasma was reduced in ELMO1 knockdown cells, even with CXCL12 stimulation (×200). (d) In HepG2 cells, no significant difference in plasma membrane-associated ELMO1 fluorescence were observed following siNPM1 knockdown cells (×200). One-way ANOVA, *p* > 0.05.

The identification of novel proteins interacting with NPM1 using mass spectrometry was used to determine the potential molecular function of NPM1 in HCC. Previous studies have indicated that the dedicator of cytokinesis (Dock180) and ELMO1 as GEF are useful in small G-protein Rac activation, which contributes to actin polymerisation to drive tumourigenesis and aggression [12]. First, high levels of exogenous NPM1 and ELMO1 expression were successfully obtained by transfecting HepG2 cells with GV141 Flag-ELMO1 plasmids and stimulation by CXCL12. We also confirmed using immunoprecipitation (IP) analyses that NPM1 is linked with ELMO1. Subsequently, the cell lysates were subjected to precipitation using an anti-FLAG antibody, and the precipitates were analysed by SDS-gel electrophoresis and autoradiography. We further confirmed that NPM1, which functions as an ELMO1-interactive molecule in HepG2 cells, was pulled down by the Flag-mediated IP using the NPM1 antibody. Co-IP results validated that NPM1 was associated with ELMO1 rather than with the control (Figure 4a). We next examined the subcellular colocalization of NPM1 and ELMO1 using an immunofluorescence assay. Upon CXCL12 stimulation, ELMO1 was mainly translocated to the plasma membrane and may transport NPM1 from the nucleolus to the cytoplasm (Figure 4b). The ELMO1 knockdown reduced the NPM1 translocation across the cell cytoplasm and nucleoplasm, even after CXCL12 stimulation (Figure 4c). However, NPM1 knockdown cells had no significant effect on the membrane enrichment of ELMO1 (Figure 4d). Therefore, NPM1 may be a critical factor for the CXCR4 chemokine receptor-induced accumulation of ELMO1 at the plasma membrane.

### 3.5 High expression of NPM1 in HCC tissues

**Figure 5:**
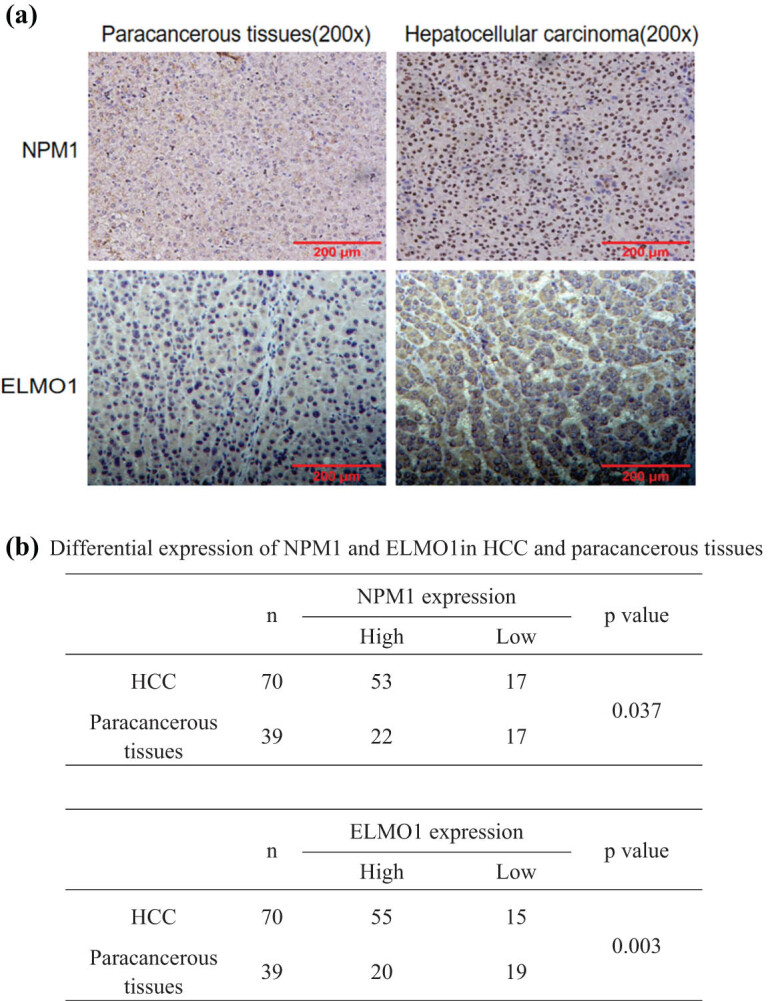
NPM1 and ELMO1 expression was significantly upregulated in human hepatocellular carcinoma tissues compared with paracancerous tissues. (a) Tumour sections for the expression of NPM1 and ELMO1 proteins were analysed in HCC tissues by immunohistochemistry with representative images shown. The representative photographs and quantification are shown (×200 magnification). (b) Differential expression of NPM1 and ELMO1 in HCC and paracancerous tissues.

To clarify the relationship between the expression of NPM1 in HCC tissues and clinicopathological characteristics, we examined the expression levels of NPM1 and ELMO1 in HCC and paracancerous tissues using immunohistochemistry analysis. The immunoreactivity of the NPM1 protein was predominantly located in the cell nucleus and cytoplasm; the immunoreactivity of the ELMO1 protein was mainly located in the cell membrane and cytoplasm (Figure 5a). Moreover, the positive expression rate of NPM1 and ELMO1 proteins in the HCC tissues was higher than that in paracancerous tissues (Figure 5b). Subsequently, we further analysed the correlation between NPM1 expression and the clinicopathological features of patients with HCC to investigate the significance of NPM1 expression in HCC. We observed a strong positive correlation between NPM1 protein levels and lymph node metastasis (Table 1). The above studies confirmed that high NPM1 expression might be a poor prognostic marker in patients with HCC.

**Table 1:** Correlation between NPM1 expression and clinicopathological characteristics

Variables	NPM1 expression		Total	*p* value
	Low	High		
Age (year)				
≤50	7	18	25	
>50	10	35	45	0.589
Sex				
Female	5	12	17	
Male	12	41	53	0.571
Tumour size				
≤5	5	20	25	
>5	12	33	45	0.533
TNM stage				
I/II	10	31	41	
III/IV	7	22	29	0.981
Lymph node status				
N0	3	30	33	
N1/N2	14	23	37	0.005

The statistical analysis of the correlation coefficient was indicated using.

Chi square test. **p < 0.05.

## 4 Discussion

In this study, we demonstrated that an increase in NPM1 expression is linked with HCC progression and may serve as a potential marker for the prognosis of patients with HCC. Moreover, a chemokine receptor-interacting signalling pathway has shown that under CXCL12 stimulation, the function of NPM1 interacting with ELMO1 promotes the migration and chemotaxis in the HCC cells. Additionally, DMF synergistically suppressed HCC progression through the NPM1/ELMO1 signalling pathway, which suggests that simultaneously targeting NPM1 and ELMO1 for HCC metastasis treatment is a promising approach to increase its therapeutic effects.

Both NPM1 and ELMO1 have been implicated in the progression of various malignancies in humans. NPM1 acts as an oncogene and a tumour suppressor in tumourigenesis, depending on the cell type and the expression levels of proteins [32]. In contrast, ELMO1 is known to utilise ligand-dependent co-stimulation to modulate cell chemotaxis and migration. However, their physical and functional interactions with each other and/or other pathways are presently unknown. In addition, although intercellular communication between tumour cells and the CXCL12/CXCR4 signalling pathway has been widely studied in tumour chemotactic and metastatic processes in many cancer types, few studies have focused on the effect of NPM1 in CXCL12-mediated HCC chemotaxis and migration. First, we functionally validated the role of NPM1 as a tumour promoter. This study’s results showed that the expression of NPM1 was positively correlated with cell mobility in HCC. Compared with the negative control group, the NPM1 silencing cells inhibited CXCL12-mediated migration, chemotaxis, proliferation, and invasion of HCC cells. In addition, CXCL12 and CXCR4 ligand receptors lead to receptor dimerization and cellular actin cytoskeleton polymerisation, which are crucial for chemotaxis and metastasis. Therefore, the NPM1 pathway is vital for the increase in cell migration ability.

Additional study analyses should be further performed to precisely verify the interactions of the pathologically relevant NPM1 with other proteins. Furthermore, our study of pull-down followed by mass spectrometry and co-immunoprecipitation showed that NPM1 interacted with ELMO1, associating with DOCK180, which formed a stable NPM1/ELMO1/DOCK180 complex and CXCL12 stimulation promoted ELMO1-mediated nuclear translocation of NPM1. When ELMO1 was knocked down by siRNA transfection in the human HCC cell line HepG2, NPM1 nuclear translocation was significantly reduced by the CXCL12 stimulation. This result showed that ELMO1 is essential for NPM1 translocation to the plasma. Simultaneously, our data show that CXCL12 directly binds to its receptor CXCR4, which constitutes the chemokine/receptor axis that impacts the downstream genes of NPM1 signalling activation. Subsequently, NPM1 translocated from the nucleus to the cytoplasm and plasma, where it interacted with ELMO1, activated small GTPases (Rac1), and ultimately triggered actin polymerisation and liver cancer cell migration. In addition, compared with the above results *in vitro*, from NPM1 expression analyses, the experiment also showed similar results *in vivo*, such that the NPM1 protein level was relatively highly expressed in the metastatic HCC tissues. This result shows that NPM1 plays a critical role in HCC metastasis *in vivo*.

HCC is one of the most devastating tumours, with high mortality and incidence rates in developing countries, especially China. Currently, there are few available treatment options for patients with advanced-stage HCC, and their prognosis remains poor [33]. Therefore, our study provided insight into a novel therapeutic strategy for patients with HCC. Notably, DMF, which is inexpensive and easy to obtain, is a commonly used industrial compound obtained from the esterification of fumaric acid and ethanol. Several studies have demonstrated that DMF has a strong cytotoxic effect *in vitro* in many cancer cell lines [34,35]. Therefore, our results suggest that DMF plays a role in inhibiting tumour proliferation and migration, which may consequently lead to tumour cell apoptosis induced by DMF. However, the exact mechanism by which DMF treatment affects HCC tumour is still unknown. Therefore, an in-depth understanding of the underlying molecular mechanisms of HCC treatment with DMF should be explored further. This study demonstrated that the NPM1/ELMO1 pathway is efficiently downregulated in HCC after DMF treatment. NPM1 signalling also regulated tumour cell proliferation, migration, chemotaxis, and invasion. In addition, our data showed that DMF significantly inhibited the process of chemotaxis and migration, as indicated by the decreased levels of NPM1 and ELMO1 protein. Summarily, these data indicated that DMF is a potential therapeutic drug for treating HCC.

In conclusion, NPM1 expression was increased in the HCC cells, and NPM1 overexpression promoted CXCL12-mediated HCC cell proliferation, chemotaxis, and migration. We also identified NPM1 as a novel factor that interacts with ELMO1 to promote metastasis. However, several challenges remain unresolved regarding the detailed interaction between NPM1 and ELMO1. Particularly, evidence from *in vitro*, *in vivo*, and clinical data analyses suggests that ELMO1 may be a potential biomarker for HCC prognosis. Finally, further inhibition of NPM1 and related signalling networks may help reverse poorly differentiated HCC. This result may provide a novel therapeutic strategy for HCC.
